# Facts from Correspondents

**Published:** 1846-11

**Authors:** 


### FACTS FROM CORRESPONDENTS.

---

### CASE OF PREMATURE DEVELOPMENT OF THE MAMMARY GLANDS.

We are indebted for the following facts to Dr. JOHN DAWSON, of Jamestown, Ohio:

To-day, (July 30th,) Mrs. M. presented her daughter to me for an opinion as to its condition. The child is a female, nearly three years of age. It has had good general health since its birth; is of light complexion, with fair hair, and rather dark eyes; is of about the usual stature of children of its age; and is the only child the mother has ever borne. Some time since, its *mammary glands* commenced being developed, and they are now about the size of large oranges, apparently well proportioned, both of the same size, with *nipples* similar to those of young ladies at the age of puberty. Neither by inquiry of the mother, nor by my own examination, could I detect any other premature development, either of mind or body.

### CONGENITAL DROPSY—(ASCITES.)

This case was that of a female child nine weeks old. I saw it October 25th, 1844, when the mother informed me that, at its birth, the abdomen seemed to be unusually protuberant, and that shortly afterwards it became subject to paroxysms of restlessness and crying, for which anodynes were administered without producing any relief. At my examination the general system was not emaciated, nor was there any anasarca of the extremities. The skin was rather soft and moist. It sucked heartily; had more thirst than natural, and had a slight coat upon the tongue. The abdominal tumor was so great as to extend down over the pubis and also upwards and backwards over the ensiform cartilage and false ribs.

I prescribed diuretic and purgative medicines, and directed iodine ointment to be rubbed on the abdomen twice a day. No amendment followed this prescription. The child fell into the hands of another physician, who tapped it and drew off a considerable quantity of water. It eventually, however, died.

### ATROPHY OF THE ABDUCTOR INDICIS MANUS MUSCLES.

This affection occurred to a man near the meridian of life, healthy, and a farmer by occupation. As well as he could remember, there has been no symptom whatever of disease of the hands previous to the time he observed the muscles to be diminishing in size, which took place about one year since. At that time an *atrophy* commenced in the *abductor indicis manus muscles* of both hands, and has continued until the present, when the space between the thumb and index finger occupied by these muscles seems to be completely vacated, giving to this part of the hand a sunken appearance. In the parts there has been no pain at any time, not even the sensation of weakness. The patient's general health has also been good during the progress of the affection, with the exception of a slight attack or two of intermittent fever.

Among other causes giving rise to partial atrophy, we commonly reckon inactivity of a part, disease of the principal nerve, pressure upon the principal artery supplying the part with blood, or chronic inflammation. Directly, or indirectly, when either of these causes appears to be in operation, they are connected with a deficient supply of arterial blood to the part. In the case before us, the trouble seems to be very obscure, no general or local disease of any kind being present; besides, it could not have originated from want of exercise, as the patient has all the time been engaged in active labor with his hands.

### A CASE OF UNCOMMON ACUTENESS OF THE SENSE OF VISION.

There is living in this region a young man of 23 or 24 years of age who is reported as being able to see with his natural eye *animalculæ* in common well and spring water. This faculty was noticed when he was some 15 or 16 years of age, by persons for whom he was at work, in consequence of his refusing very often to drink water handed to him, in which nothing could be discovered by common eyes. I made some experiments with him, enough to be satisfied that his case was no hoax; and did intend to have made more, but have lost sight of him, and suppose he has left the neighborhood.

His complexion is fair, temperament sanguine, eyes blue, less than the common size, with very small pupils.

### THE WEATHER.

The heat of this region at several periods during the months of July and August has been intense—more so decidedly than has been experienced for a number of years. The mercury in Fahrenheit's thermometer on several days in July went up to 94°, and on one as high as 96° in the shade. During the past winter we had some weather colder than has been experienced for several years. What effect these extremes of temperature may have upon health has yet, in part, to be observed. It was the remark of an old observing physician of this State, that severe winters, as a general rule, were succeeded in the spring with more than the usual amount of inflammatory diseases. From the effects of the intense heat we have recently had, I have, as yet, observed nothing in the health of a character unusual.

Jamestown, August, 1846.

